# Academy of Stomatology

**Published:** 1899-08

**Authors:** 

**Affiliations:** Academy of Stomatology


### ACADEMY OF STOMATOLOGY.

A regular meeting of the Academy of Stomatology was held on Tuesday evening, March 28, 1899, at the rooms of the Academy, 1731 Chestnut Street, with the President, Dr. M. H. Cryer, in the chair.

A paper entitled "Gout" was read by Jay F. Schamberg, M.D., of Philadelphia.

(For Dr. Schamberg's paper, see page 424.)

### DISCUSSION.

*Dr. Kirk.*—The more I study the subject the less I feel I really know about it. The presentment which the essayist has made of the conflicting opinions as to the etiology of gout is an index of the present status of the subject. One may read Haig, Luff, Garrod, and a whole list of classics on the subject, and rise from the task in a state of mental confusion. It seems that each investigator contributes some actual facts based upon experiment and observation which go to the building up of our knowledge of this exceedingly complex subject. It seems to me that the thinkers about gout have largely been endeavoring to fit certain groups of symptoms to definitions, just as we have been endeavoring to fit a certain group of symptoms to the definition of pyorrhœa alveolaris. This has led to confusion.

There were many interesting, suggestive points raised by the essayist. I shall not attempt to go over them in full, but would recall his definition of the gouty condition. If it fits the disease in so far as some of the tissual reactions to the irritant uric acid or sodium urate or other salts of uric acid are concerned, then he would possibly exclude from the definition of gout a group of allied disorders. Irritants in the blood-stream—not uric acid, but certain other toxic substances—may be regarded as producing their effects in a strictly analogous way. Though their effects would not be clinically identical, yet they may be grouped as analogous disorders, as, for example, the group of disorders produced by the condition known as auto-intoxication, or the group of disorders produced by the presence of certain waste-products of nutrition other than uric acid in the blood-stream. Having this variety of sources of irritation, we have a correspondingly large variety of diseases due to tissual reactions to these several irritants. I have held that *pyorrhœa alveolaris* is directly related to such a condition. I have seen typical pyorrhœa alveolaris in the same mouth with gouty abscess, as described by Professor Darby, and in the mouths of individuals who were typical illustrations of uric acid diathesis; and because of the frequent association of those conditions, I have been confirmed in my belief that they were, in some way, closely related in etiology, and, acting on that belief, I have in a number of instances suggested and carried out the anti-gout treatment with very marked success in the relief of the oral condition. I quite agree with the essayist that the oral manifestations of the uric acid diathesis are of the utmost importance, not alone to dentists, but to medical practitioners, though few recognize the importance of these oral manifestations as pathognomonic of gout or lithæmia. The oral conditions usually precede the other acute manifestations of uricacidæmia and act as danger signals which should enable the intelligent physician to ward off a more serious outbreak.

As to the dietetic phase of the subject, I may say that I have seen this disease manifest itself in two distinct types of individuals, —the plethoric and the anæmic. Whether the anæmia and plethora were produced as results of the diathesis I am not prepared to say, but, nevertheless, excess of uric acid was manifest in both types. I have followed out Haig's idea of interdicting the use of butcher's meats in all cases, and have found that the anæmic types do not do so well when the highly nitrogenous food has been interdicted as they do when a proper allowance for the conservation of the nutrition of the economy has been made.

The essayist has indicated, but not emphasized as much as I had hoped he would, the depressing factors which lead up to gouty attacks. The dietetic side, important as it is, I believe to be less important than the one general principle that anything that results in a large expenditure of nervous energy, as, for example, overwork or insufficient sleep, or both combined, or anything that disturbs the proper nutritional standard of the economy, is a predisposing cause of these gouty outbreaks.

I should like to talk at length on the question of oral manifestations of this systemic condition, but it is a matter that cannot be satisfactorily dealt with in one evening. The matter is still under investigation, and is engaging the thought and attention of a number of men, and I hope that in the near future we shall have more positive results of investigation to settle this important question of the relation of the gouty diathesis to the necrotic diseases of the peridental membrane.

*Dr. Darby.*—I was interested in what Dr. Kirk said about Dr. Haig's work. I hoped that I should have gotten some little comfort, perhaps instruction and benefit, from it, but I believe with Dr. Kirk that the prevention of gout, as described by Dr. Haig, does not work as well in practice as in theory. I do not think that it is so much what the man of a gouty diathesis eats as it is the quantity he eats, not so much what he eats as what he assimilates or does not assimilate, that gives him the gout. Some gouty subjects eat with impunity what others cannot eat. I myself have tried everything and done everything, have drunk from all the springs in this country and in Europe, and I do not know that anything has done me as much good as moderation in diet. Mark the word, *moderation!* I do not know that I have been any better when I have abstained from butcher's meat than when I have taken it in small quantities. While fish is recommended, there are gouty subjects who cannot digest fish; it is almost poison to them, and they had better let it alone.

Every gouty person, like every one who has not gout, ought to learn what he can eat with impunity and the quantity, and eschew the things he cannot eat and eschew the quantity he cannot digest and assimilate.

So far as waters are concerned, I think that a good spring water, as Dr. Schamberg has suggested, a gallon a day of it, is as good as any of the lithia waters or anything else in the way of spring waters. I have twice been to Carlsbad and drank very freely, and have twice taken the "cure" there. I do not know that I received any more benefit from that cure than I would had I stayed at home and filled my stomach with a large quantity of good pure spring water, such as Poland water. What a man needs who leads a sedentary life, such, for instance, as we lead, is water and pure oxygen, or its analogue atmospheric air. With these he will have as good a remedy for gout as any body can suggest.

I am obliged to Dr. Schamberg for his paper.

*Dr. Schamberg.*—I recognize that within the brief confines of a twenty-minute paper it is impossible to treat of such a broad subject as gout in the way it should be treated. One is obliged to pass over a good many phases of the subject in entirely a too cursory manner and omit some features which might properly be discussed.

I am very much interested in what has been said by Drs. Kirk and Darby, and am in accord mainly with everything that has been said in the discussion. The mechanical theory of the production of gout is one which has not been indubitably proved; it has the great advantage of plausibility. Against the idea that uric acid is a blood-poison, we have the statement I made in the paper,—namely, that preceding an attack of gout, when the blood is surcharged with uric acid, the patient is in a state of well-being; he does not suffer from those symptoms which we find in a patient who is suffering with auto-intoxication, when the blood is full of toxines and perhaps micro-organisms.

Another point I wish to make is this: that in certain blood disorders, in leukæmia and in some of the essential anæmias in which there is a great increase of white blood-corpuscles, we have an increase of uric acid in the blood by these blood-corpuscles, and we do not know such symptoms as are seen in gout; and it is only when, for some reason, the solubility is lessened and its precipitation begun that we obtained the clinical symptoms of gout, whether in the tissues or the joints, in the various viscera or in the ligaments, bone or muscles, or other elements of the human body. It seems to me that most of the symptoms, even those of visceral gout, might be explained upon the mechanical theory; for instance, it is not beyond plausibility, that the deposition of uric acid salts on a nerve-sheath can give rise to neuralgia. Nor does it stretch the imagination to believe that asthma may be produced by the precipitation of salts and urates in the bronchial tubes, giving rise to spasm. So it seems to me that the mechanical theory is perhaps as plausible as any other that has been advanced. I agree with Dr. Kirk that the conditions have been fitted to theories, and that a great deal has been said about the value of a very limited meat diet, or the entire exclusion of meat as an article of food, and I agree with him that there are many cases of under-nutrition that require meat, perhaps in fair quantities, so that we can hold to no hard-and-fast rules. The patient must be treated as a patient, and, secondarily, the disease treated.

I had under my care, and still have, a young man who developed several epileptic convulsions. The mother had been a long sufferer from migraine, which is known to occur in gouty subjects. In addition to using the ordinary treatment for epilepsy, —the use of bromides, which is perhaps the most valuable remedy, —this boy was cut off from meat diet, at first entirely and later partially, and although most of the bromides have been cut down to extremely small quantities, this boy remains free from epileptic convulsions, and I believe that the greatest measure of good has been accomplished by a low nitrogenized diet.

I think that the "cures" in foreign countries are often overestimated. As stated by Dr. Darby, a "cure" at home is frequently just as valuable. However, a man who is near the seat of his business and can leave the leisure which he is enjoying to take up some business problem is not so apt to live under a hygienic *régime* as in some foreign spot; but I am convinced that the large use of pure spring water, moderation in diet, both meat and vegetable,—because it is possible to be afflicted with gout from an excessive use of vegetables,—exercise, and a hygienic life will do most not only to prevent gout, but also a gouty diathesis.

*Dr. Truman.*—The latter part of the paper needs some explanation. I have hesitated to speak because I do not wish to bring up the subject, pyorrhœa alveolaris. But, if the paper goes out without a protest in regard to that matter, it seems to me it is in degree an endorsement of this Academy that all pyorrhœa is caused by gouty conditions. I am not prepared to endorse that, and I therefore wish simply to enter this objection, without discussing it.

*Dr. Schamberg.*—I referred in the paper merely to pyorrhœa alveolaris of constitutional origin, and in the papers I have consulted in reference to this full credit was given to gout.

I should like to ask, while the question is brought up, whether any of the members present have found gouty pericementitis in men who work in lead? It seems to me that a larger proportion of cases should occur in men of this occupation than others, and, furthermore, it is rather surprising that there is not a greater predominance of this affection in men than in women.

*Dr. Truman.*—As far as that is concerned, my observation at the University of Pennsylvania, Dental Department, shows that we have had quite as many cases of pyorrhœa there in women as in men; I think the preponderance is rather on the side of the female than otherwise. I may say, in that connection, that the larger proportion—I have seen a vast number—have had no indication whatever of gout. I have taken a great deal of pains to inquire into the history of the cases, and I am enabled to say that a gouty diathesis has but little to do with it, and, to my mind, a large proportion is purely local.

*Dr. Kirk.*—If there is anything that I would like to see made clear to the minds of the dental profession, it is that pyorrhœa alveolaris is not a disease at all, but a symptom of disease; and it may be a symptom of a very large group of diseases. I fully agree with Dr. Truman that it should not be sent out to the world as the expression of opinion of this body of men that all cases of what are so-called *pyorrhœa alveolaris,* all cases where we have a flow of pus from the sockets of the teeth, are caused by a gouty diathesis. There is such a thing as dento-alveolar abscess. What is that but pyorrhœa alveolaris, according to the definition? And we all know very well that most of those cases are cases of septic infection, local conditions, and yet, strictly speaking, pyorrhœa alveolaris; and so there are a large number of chronic irritations resulting in necrosis of the retentive apparatus of teeth which are not necessarily associated with or caused by a diathetic condition. The difficulty lies in the fact that we have no definition of what constitutes a gouty condition. I am not at all sure that all these cases of necrotic inflammation of the retentive apparatus of the teeth are necessarily due to uric acid as a constitutional factor. I believe there are such cases. I do not hold at all to the so-called mechanical theory of the destruction of these membranes by the deposition of uric acid crystals. I believe that the blood becomes loaded down with poisonous waste-products, and we have these protoplasmic poisons carried to the peridental membrane, where they induce a process of necrotic inflammation. I think the systemic condition shows that there is something more than uric acid as the irritating factor. As Dr. Schamberg has truely said, in cases of uric acid rise in the blood we have a sense of well-being, or exhilaration, during the early stages, but that passes by and shortly is replaced by a sense of drowsiness followed by migraine. We also find associated constipation, either habitual or incidental to the attack. Constipation, I believe, is one of the factors which we find nearly always present in cases of pyorrhœa alveolaris, and when habitual is accompanied by absorption of poisonous matter from the retained fæces. I believe this is not to be expressed as altogether a uric acid infection, but as a toxæmia. These poisons are carried to the tissues of the peridental membrane, and we have cell-death. I believe it is rather a chemical than a mechanical irritation.

*Dr. Schamberg.*—It seems that it might be possible for those who come in contact with pyorrhœa alveolaris to determine with mathematical accuracy whether or not they are dealing with a condition of gouty origin. It is known that after any attack of gouty arthritis there is a deposition of urates in the tissues, and if there is an opening made after these attacks, one may obtain crystals of uric acid, thus removing all doubt. These crystals are tested chemically. It seems to me that in pyorrhœa alveolaris, where there are concretions upon the roots of the teeth, it should be possible to scale off these concretions, and by a test with nitric acid and ammonia to be able to recognize minute quantities of urates. The uric acid does not occur in the blood normally, nor in the oral cavity, nor in the saliva, so that there can be no possibility of error from that source. The mere fact that a patient has not previously had gouty symptoms, or does not present at the time gouty symptoms, does not preclude the existence of gout. There may be latent gout present. If there is a gouty ancestry, or evidence that gout is present in some member of the family, this patient may have gout later on.

*Dr. Kirk.*—Chemical and microscopical tests have been made, and a short time after the publication of Dr. Peirce's essay on this subject Dr. G. V. Black, of Chicago, published a paper in which he showed that the urates of lime and soda were a constant ingredient of salivary calculus in all parts of the mouth. I have frequently made the test in these pyorrhœal cases, both by the microscope and by chemical test, but it is confusing, because one observer finds them in pyorrhœal cases and another in all cases. When I say pyorrhœal cases, I mean those which we suppose to be of constitutional origin.

It seems to me to be conclusive proof of the relation of certain forms of pyorrhœa alveolaris and gout, that under the therapeutic test of a proper anti-gout treatment these pyorrhœal conditions that are associated with gout recover or tend to recover. They respond promptly to treatment, and I think that we are as thoroughly justified in deducing, when a so-called gouty patient recovers under constitutional anti-gout treatment, that there is a relation of cause and effect, as, when a man recovers from an obscure condition under iodide of mercury, to suppose it to be of syphilitic character.

On motion of Dr. Truman a vote of thanks was tendered to Dr. Schamberg for his interesting paper.

Adjourned.

Otto E. Inglis,
*Editor Academy of Stomatology.*

